# “And that main artery's name is life”: Ecosocial injury and resurgent care in Deanuleahki, Sápmi

**DOI:** 10.1111/maq.12902

**Published:** 2024-12-19

**Authors:** Annikki Herranen‐Tabibi

**Affiliations:** ^1^ Department of Global Health and Social Medicine Harvard Medical School Boston Massachusetts USA

**Keywords:** care, ecology, kinship, resurgence, welfare

## Abstract

Based on 28 months of ethnographic research in Deanuleahki—a river valley in Sápmi, the transborder Indigenous Sámi homeland—this article traces my interlocutors’ striving to reclaim and repair ecological and kin relations through the everyday praxis of care. I trace this striving through the unmaking and remaking of local relations of care amidst encroachment by post‐Second World War Nordic welfare states and regimes of environmental stewardship. I propose a dual conceptualization of ecosocial injury and resurgent care to account for, on the one hand, care's alienation from its social and ecological contexts; and, on the other, the intimate everyday labor of revivifying relations of kinship and belonging, and conditions of material livability, within local ecologies. This defiant and desirous politics of care carves out an opening to attend ethnographically and theoretically to both dislocation and repair in spaces of Indigenous resurgence. In conceptualizing such a politics of care, the article brings into conversation key literatures in medical anthropology and in the interdisciplinary scholarship on care and Indigenous resurgence.

## INTRODUCTION

On an overheated afternoon in August 2018, I packed groceries into the trunk of my beat‐up Volvo in front of a village store in Deanuleahki, a river valley that traverses the northernmost Finnish‐Norwegian state border in Sápmi, the transborder homeland of the Indigenous Sámi people. An elder, Állan, 1 waved me over to the bus stop across the street where he sat, cooling in the shade. I walked over to greet him and, shaking my hand, he insisted on teaching me a word. *Ohkoladdat*, he explained, described the action of reindeer calves and mothers that are inadvertently separated from one another and that search for one another. The reindeer calf, despondent, would call out to its mother, and the mother, if she heard her calf's cries, would do her all to reach it. His words dramatized by emotive gestures, he explained that the reindeer mother would even try to fight her way through a fence separating her from her calf and, if she got caught in the barrier, she would furiously kick herself free with her powerful hind legs. As I lingered, Állan regaled me with stories from his time as a young reindeer herder. In catching his breath as we parted ways, he noted that many, including his younger siblings who had been removed from the land to attend the *internáhta—*the assimilatory residential school—never led that life. Secure in the fells, tending to the family's reindeer alongside his grandfather, Állan himself had been spared the effects of that alienation.

Állan's account of the cries and the furious motion of the reindeer calf and mother reflects the rawness of loss and disconnection in intimate relations that reverberates across experiences of colonization and assimilation in Sápmi. Yet, it also suggests a defiant striving to maintain and remake ties that sustain life—and the determination to break down barriers to such sustenance. At its core, *ohkoladdat* evokes both ruptures in intergenerational bonds of care and the struggle to reclaim them. As such, it provides an allegory of a central thread in the lived history of post‐Second World War Deanuleahki that was conveyed to me by my ethnographic interlocutors during 28 months of ethnographic fieldwork in Deanuleahki between 2014 and 2018. It captures with urgency the unmaking and remaking of local ecologies of care amidst the development of Nordic welfare states and their regimes of environmental stewardship. In so doing, it offers a vivid microcosm of the social and ecological transformation of relations of care in postwar Deanuleahki.

Állan's allegory illuminates the interdependent operation of what I term *ecosocial injury* and *resurgent care*. By *ecosocial injury*, I refer to my interlocutors’ experiences of care's alienation from its ecological and social context as a consequence of decades of Nordic state practice following the Second World War. Here, I draw upon Nancy Krieger's ecosocial theory of disease distribution, which elucidates the multilevel political‐economic and political‐ecological processes of “embodying (in)justice across the life course and historical generations” (Krieger, 2021, 16). The ecosocial account of care I offer in this article interrogates the interplay of health, well‐being, and the environment by examining locally situated understandings of the relationship of care with kinship and ecology. Crucially, I extend this inquiry to encompass everyday practices of Indigenous resurgence.

Through the analytic of *resurgent care*, I in turn trace the intimate everyday practices of care through which my interlocutors strive to reclaim and remake relations of kinship and belonging, and conditions of material livability, amid rapid social and ecological change. I approach care (Sá. *áimmahuššat, dikšut, áittardit, fuolahit*) as everyday practices of tending to the intersubjective and interspecies other's health, well‐being, and survival—and of the organization of life to ensure those ends. In turn, I understand Indigenous resurgence, following Leanne Betasamosake Simpson, as embodied everyday practices of decolonization and, more precisely, as practices of regenerating community and mobilizing for systemic change and self‐determination (Simpson, 2016). Further informing my engagement with resurgent world‐making are scholarly works, political mobilizations, and protest movements supporting Indigenous self‐determination, as well as educational and therapeutic practices oriented toward Indigenous futurity and healing (Deloria et al., 2018; Gone, 2013, 2021; Grande, 2015, 2021; Kuokkanen, 2020; Martineau, 2015; Million, 2020). My engagement with resurgence through ethnography in Sápmi illuminates how my interlocutors are, in their everyday practices of care, engaged with an intimate form of (re)vitalization (Sá. *ealáskahttit*).

The analysis that follows is structured around an extended vignette that illustrates the interlocked workings of ecosocial injury and resurgent care in the life of a long‐term kin caregiver in Deanuleahki, whom I'll call Biret‐Ánne.^2^ Her narrative illuminates the radical potential and the tensions and contradictions that mark the praxis of resurgent care as the intimate labor of reclaiming and remaking social and ecological relations upon which health, well‐being, and survival depend. Here, everyday practices of care amidst ecologically grounded kin relations constitute vital sites of transformation, desire, and striving. Crucially, Biret‐Ánne's praxis of resurgent care is agentive and action‐oriented, rather than merely a matter of aspiration or disposition. In it, the intimate, intersubjective labor of care lays bare the rupturing and repair of vital ecosocial relations in the region's postwar history.

My engagement with Biret‐Ánne's narrative and caregiving praxis exemplifies the mode and methods of my ethnography. A non‐Sámi expat Finn, I worked longitudinally with multigenerational families in Deanuleahki, developing a series of interconnected care biographies through interviews in which interlocutors were invited to narrate what they viewed as significant experiences of giving, receiving, and witnessing care over their lifetimes.^3^ I paired these multilingual interviews with extensive participant‐observation in the context of everyday caregiving encounters and of subsistence activities such as salmon fishing, reindeer husbandry, trapping, and foraging. Crucially, my long‐term research in Deanuleahki saw a collaborative redefinition, under the guidance of local interlocutors and mentors, of care as the object of study to encompass kin and ecology as its key loci.^4^ I thus approach care as everyday practices of safeguarding the intersubjective and interspecies other's health, well‐being, and survival.

In what follows, I begin by elaborating on the characteristics of ecosocial injury and resurgent care as they appeared in my encounters with Biret‐Ánne. I then explore these concepts’ relationship with the interdisciplinary theorization of Indigenous care, kinship, and resurgence, and their contributions to the ethnography of care within medical anthropology.

## RESURGENT CARE AS INTIMATE PRAXIS

On a July afternoon in 2015, as cold light streamed into Biret‐Ánne's living room through windows overlooking the Deatnu river, she peeled back layers. Lifting fabric to uncover skin, she revealed a scar on her torso from cancer treatment years prior. Etched into her skin, this memory of her own illness was overlain with the bodily and emotional toll that two decades as her housebound mother's primary caregiver had taken on her. She often wondered whether, overwhelmed by the stress and exhaustion that accompanied everyday caregiving, she had delayed seeking treatment when the telltale pain first made itself known. Once Biret‐Ánne's cancer was diagnosed and chemotherapy weakened her, she was weighed down by the fear that her mother's care would be compromised in her absence. So, too, she wondered: would her loved ones now care for her in crisis, as she had done for her mother?

I had first heard Biret‐Ánne's voice one year before this encounter. I cold‐called her during my first visit to Deanuleahki in 2014 at the suggestion of others I had interviewed. Pine needles scratched my bare feet as I paced outside a newfound friend's home, and I introduced myself to Biret‐Ánne. I told her about my nascent research interest in the relationship between families and the institutions of the postwar Nordic welfare states in their northernmost reaches, and that I had heard she might have important perspectives to share. Biret‐Ánne, a plain‐spoken woman with an acerbic wit, immediately made clear she had more important work than speaking with me, a nosy stranger. Biret‐Ánne noted that her mother, Rávdná, was sleeping upstairs; as soon as Rávdná awoke, Biret‐Ánne would sprint up to care for her. As I gestured to end the call, however, she unexpectedly drew me in, suggesting that we nevertheless speak right there and then. She took charge, launching into a narrative that had me scrambling to capture her poignant words.

As Biret‐Ánne described her decades of caring labor on that first call, she stressed that it was no thanks to local officials or welfare state institutions that she had persevered as a kin caregiver. The state, she declared with palpable anger, “is pitilessly whoring us out, pitilessly exploiting our love” (Finnish [Fi.] “*huoraa surutta meidän kanssa, hyväksikäyttää surutta meidän rakkautta”*). Biret‐Ánne's inimitable expression—“pitilessly whoring us out”—captured her fury at what she viewed as the state taking advantage of her physical and affective expressions of care for her closest kin, exploiting her intimate labor for material gain while leaving her own health and material conditions precarious.

As we returned to this narrative iteratively over the years that followed, the physical and financial strain of round‐the‐clock, decades‐long caregiving was evident in Biret‐Ánne's memories, present words, and in the unspoken alike. Yet, she insisted, care was not only implicated in causing her scars. Instead, it was profoundly reparative and intimately bound up with the always‐incomplete process of tending to mental and material wounds that spanned generations and had marked her childhood and youth. As she phrased it upon my return to Deanuleahki for long‐term fieldwork in 2016:

(Fi.) *Niin paljon on tämä äidin omaishoito korjannut lapsuudesta niitä asioita. Olen saanut muistoja kaivettua joista jäi paitsi kun ei ollut kotona. Olen saanut tietää asioita omista vanhemmista ja kaukaisemmista ihmisistä. Ei sitä olisi koskaan kuullut näitä asioita jos ei olisi äidin kanssa elänyt hoitotyössä*.

Serving as my mother's family caregiver has repaired so many of those things from my childhood. I've been able to dig up memories I missed out on by not being at home. I got to know things about my own parents and about kin further away. … I would never have heard these things if I hadn't lived with my mother, working to care for her.

The potency of this work of repair did not simply lie in an individualistic or transactional practice of caring for oneself by caring for another. Instead, over our decade of conversation, Biret‐Ánne repeatedly narrated her intimate labor of familial care as the deliberate reappropriation, repair, and restoration of that which had been harmed by the ecosocial injuries of the postwar decades. Her fury at present‐day exploitation and historically grounded dislocation was coupled with her everyday moral, emotional, and embodied striving to reclaim and remake, through the labor of care, that which had been harmed by the social and ecological transformations of the postwar era. In a manner that echoes across the narratives of my ethnographic interlocutors, care itself (Sá.: *dikšu, áimmahuššan, fuolaheapmi, áittardeapmi*; Fi.: *hoito, hoiva, huolenpito*; Norwegian [No.]: *pleie, omsorg*) emerges as an analytically luminous site of everyday acts of resurgence.

As my conversation with Biret‐Ánne unfolded over time, it became clear that her outrage at the pressures of austerity and responsibilization—pressures that poured out in her characterization of the state as pimp, and that accord with anthropological analyses of care amidst neoliberal statecraft (e.g., Garcia, 2010; Han, 2012; Biehl, 2013)—reflected a longer lived history of state practice in Sápmi. In particular, understanding her narrative and everyday praxis of care required taking account of the deeper social and ecological upheavals caused by the institutional embedding of Nordic nation‐states in Sápmi through the post‐Second World War process of welfare state building.

Across Sápmi, the Second World War is marked in collective memory by occupation and physical destruction wrought by Nazi Germany in northern Norway and Finland; the traumatic large‐scale displacement of the civilian population across Sápmi; and the redrawing of state borders between Finland and Russia, leading to the enduring displacement of entire Skolt Sámi communities (see Lehtola, 1994, 2012, 2015). Following the war, rapid infrastructural and institutional development brought roads, schools, and clinics to Deanuleahki, cementing the intensification Finnish and Norwegian state presence in this border region. Such intensification, I argue, centered on the state‐driven institutionalization of care within the archetypal institutions of the welfare state—health care, social service, and public education institutions. This institutionalization figured prominently in Finnish and Norwegian postwar state‐building and in Sámi communities’ integration into Nordic nation‐states. While the postwar Nordic states form an oft‐celebrated success story globally—one marked by socialized medicine and advances in public health, and by high‐achieving education systems—these institutions figure with equal prominence in experiences of ecosocial alienation and distress among my interlocutors in Sápmi. Indeed, many among my interlocutors depict Indigenous Sámi relations of care as having been increasingly “constrained” (Sá. *gáržžiduvvon*) and their economic independence curtailed by societal pressures related to the expansion and consolidation of state presence. Here, the interlocking institutional domains of health, social welfare, education, and ecological stewardship (such as livelihoods management, forestry, and conservation regimes) appear in my interlocutors’ narratives as alienating intergenerational care relations from their social and ecological contexts—an alienation I term ecosocial injury.

Biret‐Ánne is part of to the residential school generation that had experienced the most aggressive effects of postwar assimilation in Finland (c.f., Rasmus, 2008; Ranta & Kanninen, 2019, 145–71). She often spoke of being forced to leave her childhood home for schooling as a young child, and the ensuing familial and cultural alienation that had marked her life. Central to this alienation was what she described as the loss of language. The school years had, she argued, “sat my mother tongue on the shelf…stagnating as the language of a child” (Fi. “*äidinkieli pantiin hyllylle istumaan*…*jäi lapsenkielen tasolle”*): she neither spoke nor heard North Sámi in that institutional environment. Caring for Rávdná had allowed her to break this stagnation, giving her “the possibility of advancing with [her] mother tongue” (Fi. “*mahdollisuuden kehittyä äidinkielessä*”). In her telling, especially during times of serious illness and decline, language had drawn them close, filling the space carved out by pain.

Crucial here was the dynamism of that which was being remade: “the possibility of advancing” rather than a static identity or a fixed set of attributes. Indeed, as Biret‐Ánne noted in reference to her family's language and livelihoods during the separation imposed by the residential school years: “Neither they nor I had lived in naphtalene [i.e. mothballs]” (Fi. “*Eivät ne enkä minäkään ole missään naftaliinissa eläneet*”). There was no reclaiming culture or ecosocial relations as a fixed, immutable entity; rather, both were always in motion.

To Biret‐Ánne, language itself reverberated with such dynamism. As she described restoring North Sámi as her mother tongue, she emphasized that caring for Rávdná had allowed her to incrementally recover the fuller dimensions of her stunted language. This was particularly pronounced with respect to expressions of relationship to time: an impoverished language shaped by the grammatical and lexical influences of the Nordic languages, she argued, “lacks a look back to where I've come from, where I'm going” (Sá. “*das váilu geahčastat maŋos, gos mun lean boahtán, gosa mun manan*”). To her, this accentuated the significance of linguistic forms that position actions in time, such as the North Sámi verb suffix “‐*goahtit*”—to begin to do something—and the verb *šaddat*—to become or grow into something. She saw such linguistic forms as reflecting a recognition of interconnected history and an interdependent future, and therefore as vital to the linguistic and relational webs she was remaking through caregiving.

Over the course of my long‐term fieldwork in Deanuleahki, Biret‐Ánne and I grew close, and I spent countless days and nights in her home, observing and supporting her everyday practice of caregiving. Once, as we walked through nearby woods, Biret‐Ánne described her daily routine. She would wake up after five in the morning, never sleeping through the night in her two decades of caregiving. Likening her nights to those with a newborn child, she explained that Rávdná might call out or bang on the walls in distress or confusion; Biret‐Ánne would wake twice a night of her own accord to bring Rávdná water. She would prepare Rávdná’s medication and breakfast; she would then attend to toileting, powdering, lotioning, and diapering Rávdná, before setting her down to nap. She would prepare lunch, then dinner; once again, Rávdná would rest. Rávdná would take her evening medication, then bathe, and Biret‐Ánne would brush her teeth and put her to bed. Once Rávdná was asleep, Biret‐Ánne had a few hours to herself; her last step, before going to sleep herself, was to bring Rávdná water to keep her mouth and throat from dehydrating.

When staying with the family, I would assist in these daily tasks when I was invited to do so: helping lift Rávdná into her wheelchair or layering on woolen clothes and blankets on her to keep her warm; tending to the foam‐covered headphones through which a Sámi‐language radio station blared directly into her ears; and the ever‐necessary tasks of food preparation, cleaning, and laundry. At times, I would simply sit by Rávdná’s bedside. By then limited in her vision and hearing, she would determinedly hold onto my hand and ask me whose daughter I was, whether the weather outside had changed, verify what time of the year it was, or inquire whether I had been to her beloved places in the fells. As Biret‐Ánne emphasized, Rávdná’s questions weren't idle chatter: her mother had lived off the land and experienced firsthand the importance, for survival, of respecting and closely observing the local ecology.

Integral to Biret‐Ánne's reclamation of language and of her parental relationship with Rávdná was the knowledge of kin. Even as Rávdná’s illness progressed and strength waned, she continued to narrate her life, entwining it with those of Biret‐Ánne's grandparents. Learning of kin close and distant alike, and the lands and waterways they had inhabited, animated the repair in which Biret‐Ánne was engaged. This knowledge, she said, was at the core of what Rávdná had given her during the decades of caregiving; it had sustained her even amidst the most physically and emotionally taxing phases of giving care.  In caring for her mother, Biret‐Ánne thus engaged in caring for intergenerational memories in defiance of their erasure.^5^ Indeed, she insisted on the wider societal significance of her intimate praxis of taking back and building anew vital ecosocial relations.  In caring for Rávdná, she saw herself as revivifying forms of reciprocal care from which she had been disconnected in her childhood and youth.

The local ecology of Deanuleahki was central to these reciprocal forms of care, and to the alienation thereof. Biret‐Ánne's family and extended kin had for generations lived from reindeer husbandry, herding sheep, and fishing. As she recounted, children and youth were cared for collaboratively by kin in the context of these subsistence activities, including by cultivating their active participation in fishing and in foraging for cloudberries, bilberries, and lingonberries. This was intergenerational care as the reciprocal sharing of skills.

However, the war had marked a point of rupture for her family's place within this ecology. Biret‐Ánne's father, having fought in the Finnish forces on the northern front, carried the wounds of war into the family's livelihoods. The sequelae of wartime injuries left him unable to participate in reindeer husbandry.

Fishing on Deatnu became a key locus of Biret‐Ánne's shared experiences with her parents, siblings, and grandparents, and was particularly significant as a source of uninterrupted moments with her disabled father. Her mother would pack her children into the fishing boat with enough water, bread, layers of warm clothes, and blankets to spend hours upon hours on the river—resembling, Biret‐Ánne said, a merganser hen with its sprawling, floating brood. The grayling and salmon they caught were materially necessary for feeding the multigenerational family, with the bounty variously boiled, fried, salted, smoked, and stored in the cellar.

Learning to pole and row the family boat, and to observe the river and the species of fish and birds to which it was home, was here part of a wider multisensorial learning environment in which, as a child, Biret‐Ánne could feel, listen, and look for that which sustained life. Could the hay she held in her hands insulate homemade reindeer hide shoes for the winter? Which tree's bark ought she collect for treating reindeer skin? Was the salmon she had caught mature, or so young that it should be allowed to plunge back into Deatnu and return to feed the family the following year?

The residential school years, however, severed Biret‐Ánne from this ecology. Returning home for a week or two at a time during school breaks did not allow enough continuity for learning vital livelihood skills. Indeed, as her father's disability required her mother to tend to the family's reindeer stock, Biret‐Ánne saw her mother vanish into the fells for long stretches of her early childhood. But just as Biret‐Ánne grew enough that she might have joined her mother in the fells, her years in the residential school tore her away. Being removed from her home environment and intergenerational relations, she could not learn the skills and knowledge that accompanied daily and annual cycles of subsistence activities. As she recounted, profound alienation resulted from the diminishing scope of shared experiences wrought by this institutionalized intergenerational separation.

In Biret‐Ánne's telling, language and relational knowledge were intimately connected to the livelihoods and to the labor of mutual sustenance in the multisensorial environment from which the residential school years had removed her. As we sat drinking tea in her kitchen at night after Rávdná had fallen asleep, Biret‐Ánne would often share joyful memories of such labor. Of washing clothes with her grandmother on the shores of Deatnu, with a vat of hot water and washboard for her grandmother, and the two of them wading into the stream to rinse the clothes—playing, splashing, clothes swirling in the powerful stream, Biret‐Ánne lunging to catch what her grandmother sent her way. Of helping, as a small child, to sew together walls for reindeer herders’ *lávvu* (a temporary dwelling supported by wooden poles and walls made of reindeer hides or fabric), at times hiding underneath them to pretend she was in a *lávvu* of her own. Of tarring boats with her mother, the scent of tar still reminding her of stories told amidst shared, reciprocal labor.

Away at residential school, she lamented, she did not learn *duodji* (traditional craftsmanship that encompasses weaving, sewing, leathercraft, woodworking, and bone carving): she never learned to make *nuvttohat* (traditional reindeer skin shoes) or to *neaskit* (soften reindeer skin by scraping connective tissue). Indeed, her homemade reindeer hide shoes were often mocked by Finnish students and school staff, and her attempts at sewing at school only received scolding from teachers for the mess she made. The few times she returned home from school, the adults were too busy with their everyday labor, and some later too frail, to teach her. This disconnect was compounded by the ever more restrictive regulations that continued to make the practice of subsistence livelihoods more inaccessible and unaffordable to many in the community.

The ensuing sense of alienation would propel Biret‐Ánne away from Deanuleahki for decades: she only returned when Rávdná’s deteriorating health made her previous living arrangements untenable. In caring for Rávdná, she became determined to remake the senses and skills whose potentiality she had experienced in early childhood but from which she had been severed in her school years. Biret‐Ánne's presence by her mother's side through Rávdná’s long illness transformed her understanding of the local land and waterways, populating it with stories of long‐gone kin and of Rávdná’s lifetime of tending to the reindeer, fishing, and foraging—stories with which Rávdná continued to find her place in the world even on her final ambulance journeys.

Crucially, to Biret‐Ánne, learning through *duodji* was intimately connected to a sense of shared responsibility for everyday needs that, in the harsh physical environment of the Arctic, could easily become a matter of life and death. To her, there was a direct sense in which storytelling and multisensorial learning through *duodji* invoked a sense of responsibility for others, and of belonging in social and ecological relations. This participation in everyday labor in the family and within subsistence livelihoods stood in stark contrast with the relationship to labor Biret‐Ánne experienced within the residential school, where work was used as a punishment by the staff overseeing the children's care. The school as a disciplinary space thus served as a site of care's transformation from relational praxis into remunerated work within a public institution. In her intimate praxis of taking back and building anew disrupted ecosocial connections, Biret‐Ánne saw herself as reclaiming the reciprocal care she had witnessed, but from which she had been severed, in her childhood and youth.

She often named her grandmother as a central node in such reciprocal webs care, one who frequently fed, clad, and housed orphaned children and others experiencing vulnerability in the community. As it became the grandmother's turn to be sustained by her younger kin, Biret‐Ánne grew from a cared‐for to a caregiver (Fi. *hoidettavasta hoitajaksi*) as a young child, helping to bathe her ailing grandmother or serving as her “subsitute for legs” (Fi. *jalkojen korvikkeena*), running and climbing to gather willow bark with which to treat reindeer skin before it was turned into clothing and household items. From such reciprocities emerged, in Biret‐Ánne's telling, a “built‐in social safety net” (Fi. *sisäänrakennettu sosiaalinen turvaverkosto*) marked by interdependency. This “built‐in social safety net” of capacious reciprocal relations of social and ecological care stood in stark contrast with the state‐provided institutional order of formal health and social services. Indeed, the workings of those institutions were in Biret‐Ánne's experience often inimical to such care:

*Sá. Olbmot leat biddjojuvvon dakkár kássaid sisa, dat eai gulahale gaskaneaset. Nuorra olbmot eai oaidne makkár olmmoš lea boarisin. Ja boares olbmot eai oažžo doarjaga. Dat gal guđđojuvvo okto, hilgojuvvo gosa nu čihkkii čohkkat áibbašit servodahkii. Mun jáhkkán ahte dáinna koansttain dat máilbmi nohká jos olbmot eai váldde vuhtii*.

People have been put inside these boxes, they no longer speak to one another. Young people don't see how people are when they get old. And old people don't get support. They're left alone, abandoned to sit in a corner and to long for society. I believe this way the world will end if people won't pay attention.

Biret‐Ánne's exhortation here is for the recognition that all beings had needed and would need care. In her analysis, the prevalent lack of such recognition was traceable to the separation of generations into the “boxes” of distinct institutional spaces, from schools to elder care facilities, in a manner that undermined practices of reciprocal care. This separation figures centrally in care's alienation from its social and ecological contexts. Biret‐Ánne, refusing this alienation through her everyday labor of care, sought to restore—and resituate herself within—vital webs of reciprocity.

One evening in 2018, near the end of her mother's life, Biret‐Ánne reflected upon ruptures, sacrifices, and acts of repair that were implicated in her decades as kin caregiver. She mused on how rapid social change in Deanuleahki's recent history had separated people from one another and from the mutual support upon which life depended. She emphasized that every day, she was caring for something that, if lost, could never be compensated.

*Sá. Goittotge dat deháleamoš, man mihkkige ii sáhte buhtadit, lea eallin. Ja olbmuid dikšun lea eallima dikšun. Giela dikšun, dat lea eallima dikšun. Mii leat dušše unna balvaleaddjit dán eallimis…. Juohke áidna lea ovtto dárbbašan nuppiid, juohke áidna bargu lea leamašan dan servodaga doarjuma dihtii. Mu mielas dat lea lahppon dálá máilmmis*.

Still, the most important thing, for which nothing can compensate, is life. Caring for people is caring for life. Caring for language, that is caring for life. We are just little servants in this life. … Every one of us has always needed others, everyone's work has been in support of society. I think this has gotten lost in today's world.

Here, the imperative to care permeates Biret‐Ánne's analysis of loss and dislocation—the dislocation, that is, of reciprocity among people and in support of society, which she stated had been “lost in today's world” (Sá. “*lahppon dálá máilmmis*”). Overriding that sense of loss is the primacy of caring for “life” (Sá. *“eallin”*) as something “for which nothing can compensate” (Sá. “*man mihkkige ii sáhtte buhtadit*”). This underscores her assertive commitment to life and lifeway, to caring and enduring in the context of ecosocial injury.

In Biret‐Ánne's narrative, then, resurgent care emerges as an intimate site of transformative, reparative praxis and social critique. The temporal dimensions of the social critique emerging from her praxis of resurgent care particularly underscore the limitations of a social order in which ties of interdependence, both interspecies and intersubjective, are frayed. She connected these observations back to Deatnu itself.  

*Sá. Mii eat leat dan eallima vuoddjit. … Dat nu gohččoduvvon fridjavuohta, dat áitá min buohkaid. Mii eat muitte ahte dat mo mii láhttet dain guollečáziin ii leat dušše dán gease dahje boahtte gease, leatgo doppe guolit jagi geahččen, viđa jagi geahččen, viđalogi jagi geahččen. Muhto dat lea min iežamet láhtten*.

*Jus mii badjelgeahččat muhtin ealli dahje luondoealáhusvuogi dahje birgenlági ohcadeami luonddus, mii badjelgeahččat iežamet. Dat Deatnu gal birge  min haga muhto mii eat birge Deanu haga. … Deatnu lea nu gohčoduvvon váldorávdnji. Ja mii leat buohkat oassi váldorávnnjis. Ja dan váldorávnnji namma lea eallin. Ja dasa mii leat vuollegasat buohkat*.

We are not the drivers of this life. … That so‐called freedom, it threatens all of us. We don't remember that how we behave with our fishing waters is not just [a matter of] this summer or next summer, will there be fish in a year, five years, fifty years. But it is [about] our own behavior.

If we treat with contempt some animal or subsistence livelihood or the search of sustenance from nature, we treat ourselves with contempt. Deatnu will survive without us but we will not survive without Deatnu. Deatnu is a so‐called main artery. And we are all part of that main artery. And that main artery's name is life. And to that we are all subject.^6^

Casting Deatnu as a life‐giving artery, Biret‐Ánne thus underscores how livability hinges on tending to and restoring relations with human and more‐than‐human kin. To be severed from such relations, she argues, threatens life itself. I shall now turn to the resonances of the foregoing ethnographic analysis with the interdisciplinary scholarship on Indigenous care, kinship, and resurgence, and with the ethnography of care within medical anthropology.

## TOWARD A RESURGENT POLITICS OF CARE

A vibrant interdisciplinary scholarship on Indigenous care, human and more‐than‐human kinship, and resurgence (Grande, 2021; Hobart & Kneese, 2020; Million, 2020; Simpson, 2016) has long reckoned with histories of inequality, violence, and exploitation affecting Indigenous peoples, while refusing to be reduced to narratives of damage and harm (c.f., Tuck, 2009; Grande, 2021; Million, 2020). My account of resurgent care amidst ecosocial injury engages with and complements this scholarship through longitudinal ethnographic analysis of intimate practices of care as vital loci of resurgence, grounded in intergenerational kin relations and ecology. Crucially, it builds upon this scholarship and advances the anthropological study of care by articulating a desirous and defiant politics of resurgent care.

I shall now elucidate the analytical intervention of resurgent care amidst ecosocial injury by engaging key scholarship on Indigenous resurgence, kinship, and care in a mutually informing conversation with existing ethnographic literature on care within medical anthropology. This conversation is structured around three key themes: (1) the radical potentiality of intimate practices of care; (2) kinship as a site of resurgence; and (3) desire and striving as everyday instantiations of resurgence effectuated through the practice of care.

1. *Radical Potentiality of Care*

In her luminous essay, “Resurgent Kinships,” Dian Million writes, “Care is a radical act in our times. It is inherent to our sovereignty, as embodied, lived experience with our relations” (2020, 392). Paralleling this declaration, Hi'ilei Hobart and Tamara Kneese offer a similarly expansive account of the political stakes and communal reverberations of what they term “radical care,” which they characterize “as a set of vital but underappreciated strategies for enduring precarious worlds” (2020, 2). Crucially, they note, radical care is “inseparable from systemic inequality and power structures” (2020, 3), unfolding as it does amidst the hard edges of historical dispossession and present‐day inequity. Yet, they underscore, this entwining does not efface care's transformative power: instead, “fragile communities operationalize care toward liberatory ends despite, through, and alongside unequal power structures” (2020, 7). Radical care, they argue, serves as a vital locus of “hope” (2020, 3) and of collective mobilization amidst inequity and dislocation—much as the praxis of resurgent care amidst ecosocial injury does in the intimate everyday domain. It is here that resurgent and radical conceptualizations of care are aligned in their recognition of the complex dynamics of power that may impinge upon practices and relations of care, and in their unyielding commitment to the radical, transformative potentiality of everyday praxis and communal mobilizations of care amidst the hard edges of inequity. Such an approach yields opportunities for the ethnography of care to contribute to what María Puig de la Bellacasa has characterized as the “reclaiming care… from tendencies to smoothe out its asperities—whether by idealizing or denigrating it” (2017, 11).

Care's entwining with power, inequality, exploitation, and neglect is likewise a vital throughline in the ethnography of care within medical anthropology. Thus, João Biehl (2013), Angela Garcia (2010), and Clara Han (2012) have offered astute warnings against the valorization of familial care. They have particularly critiqued the renewed centrality of the immediate family, wider kin, and other “intimate relations” (Han, 2012, 22) to caregiving amidst the neoliberalism‐inflected downscaling of public institutions of health and social services across the globe (Biehl, 2013, 125–32; Garcia, 2010, 187–93; Han, 2012, 46). Their ethnographies illuminate the potential of familial care to be actively harmful and infused with violence. This insight echoes Joan Tronto's observations regarding the propensity of societal transformations—such as cuts to publicly funded services and structural inequity—to place heightened demands on informal caregiving (e.g., Tronto 1993, 9–30, 109–67). Under such conditions, as Puig de la Bellacasa writes, “‘to care’ can be devouring for women and other marginalized carers” (2017, 163).

The specific moralization of families to care is reflected in the wider mobilization of publics as sources of informal care amidst cuts to welfare spending globally. Thus, Andrea Muehlebach's account of the moralization of the provision of nonremunerated care amidst the downscaling of the Italian welfare state and the particular idealization of “relational care” (Muehlebach, 2012, 133) exemplifies the potential of caregiving's moral stakes to be harnessed in an exploitative manner in service of neoliberal state practice. These ethnographic perspectives underscore the risks of, in Puig de la Bellacasa's terms, “[entangling] care with hegemonic regimes” (Puig de la Bellacasa 2017, 9)—be those regimes neoliberal, colonial, racialized, and/or gendered.

This recognition of the varied “appropriations of care” (Puig de la Bellacasa 2017, 10) in the service of power has led to calls for scholars to “embrace [care's] ambivalent character” (Puig de la Bellacasa 2017, 11; see also Murphy, 2015; Duclos & Criado, 2020). A recognition of such ambivalence—or, perhaps more appropriately, multivalence—connects the analytic of resurgent care put forth in this article with the expansive ethnographic literature on care within medical anthropology and with wider theorization surrounding care's radical potential.

2. *Kinship, Colonization, and Resurgence*

The analytic of resurgent care centers the intimate practices of reclaiming, remaking, and rebuilding relations to kin, language, and ecology through the everyday labor of caregiving. The wider significance of kinship as a site of colonization and of resurgence emerges powerfully from the Indigenous scholarship on care. Thus, Sandy Grande argues, the multilevel violences against Indigenous peoples over generations of colonization specifically assailed “expansive relations of making kin” (Grande, 2021, 46) in realms spanning religious practice, child rearing, land, and language alike. Scholars of Indigenous resurgence have thus underscored the significance of human and more‐than‐human kin‐making for interrupting and repairing such harms, with Grande framing “care as a praxis of relationality and kinship” that animates “the purposeful building of coalitions and networks of human and other‐than‐human kinship” with profound decolonial, anti‐imperial, and anti‐capitalist significance (Grande 2021, 46; see also Marley, 2020, 44; Million, 2020, 396–99; Simpson, 2016). Notably, Million argues that such practices of relationality—ones reflecting “embodied, affective existence and spiritual, life‐giving relations to both humans and non‐humans”—are vital to Indigenous health, well‐being, and resurgence (Million, 2020, 396).

Such agentive kin‐(re)making animates practices of elder care, child rearing, and interspecies subsistence activities among my interlocutors in Sápmi. Attending to more‐than‐human ecologies of care—and to the agentive remaking thereof—is crucial to examining the ecological dimensions of care with contextual specificity. The analytic of resurgent care amidst ecosocial injury thus echoes and reinforces the framing of kinship as a site of rupture and repair in interdisciplinary Indigenous scholarship on care and resurgence. Indeed, instead of formal initiatives and public mobilizations—such as protest movements, cultural gatherings, therapeutic programming, or the institutionalization of Indigenous self‐governance (see, e.g., Martineau, 2015; Gone, 2013, 2021), my conceptualization of resurgent care underscores what Leanne Betasamosake Simpson has framed as “intimate resurgence” among kin (Simpson, 2016, 1; see also Barker et al., 2021). As intimate praxis and cultural critique, resurgent care draws upon and reinforces locally emergent mobilizations for Indigenous futurity (*sensu* Deloria et al., 2018). It does so by centering everyday practices through which my interlocutors seek to ensure the intersubjective and interspecies other's—and their own—health, well‐being, and survival.

The disruption of Arctic Indigenous kinship ties through state intervention has previously been examined in the register of care within medical anthropology in Lisa Stevenson's influential account of Canada's post‐Second World War regime of “biopolitical” and “anonymous care” among the Inuit (Stevenson, 2014, 5). Such state‐provided care has, in Stevenson's analysis, pathologized the Inuit as the objects of ostensibly benevolent interventions and thus been integral to the colonial consolidation of Canada's postwar sovereignty in its northernmost reaches (Stevenson, 2014, 28, 78–85). Stevenson notes how such measures of care by the Canadian state have often prioritized “surveillance” and “preserving life” in a manner that is “indifferent” to the context, person, place, and time. Stevenson situates her analysis of the erasure of Inuit needs, priorities, and sociocultural and historical context amidst “welfare colonialism,” a term first used by Robert Paine to characterize the adverse effects of the consolidation of post‐Second World War Canadian state sovereignty on Inuit homelands and the creation of Indigenous dependency on state institutions (Paine, 1977, 7–28). With the expansion of health, education, and social service provision undertaken in parallel with policies that jeopardize Indigenous self‐governance as well as cultural and subsistence practices, welfare colonialism has proven a resonant analytic across the Circumpolar Arctic (Jull, 1999; Kuokkanen, 2009; Wachowich, 2006). Crucially, while many analyses of welfare colonialism have focused narrowly on institutions of health and social welfare, my ethnography illuminates how, following Rauna Kuokkanen, this mode of state practice centrally involves the “radical undermining of traditional livelihoods” (Kuokkanen, 2009, 111).

This more expansive understanding of welfare colonialism coheres with my conceptualization of ecosocial injury as effectuated through wide‐ranging regimes of state‐sanctioned care. By tending to such injuries, resurgent care constitutes a potent response to the ecological and social alienation of care relations. The dual analytic of ecosocial injury and resurgent care thus contributes to the examination of disrupted kinship ties within the medical anthropology of care by foregrounding the affirmative commitment to remaking the “expansive relations” (*sensu* Grande, 2021, 46) upon which health, well‐being, and survival depend (see also Million, 2020, 400–03). It is to this ecologically grounded, agentive mode of care that I now turn as the defining characteristic of the intimate praxis of resurgent care.

3. *Desire and Striving as Everyday Instantiations of Resurgence*

Recall the ethnographic encounter with which this article began: Állan's word, *ohkoladdat*, and the allegory contained therein. In his narrative, the reindeer calf and reindeer mother's striving for both freedom and connection contains ecological and intergenerational dimensions and takes place against the backdrop of externally imposed separation, alienation, and rupture. As Biret‐Ánne's intimate praxis of resurgent care illuminates, such striving is often incomplete, contradictory, and itself riven with pain and loss, yet holds radical reparative power. Likewise, the word *ohkoladdat*, as taught to me by Állan, emblematizes the striving by my interlocutors to remake lives and lifeways life amidst their increasing precarity. This is care in a defiant key: emerging from a field of contestation and reflecting an assertive commitment to enduring and acting upon the world, a refusal to come undone. This makes for a radically different “alternative politics of care” (Stevenson, 2014, 15–16) than the mournful, melancholic, and elegiac analyses offered in prior anthropological scholarship (e.g. Garcia, 2008, 2010; Stevenson, 2014).

Indeed, my interlocutors’ narratives center on an assertive commitment and striving to ensure the vitality of ecologically and relationally situated practices of care. This focus aligns with Eve Tuck's proposal of deploying an analytic of “desire” to interrupt both the prioritization of “damage‐centered narratives” and the denial of colonial wounds in social scientific analyses in Indigenous lives (Tuck, 2009, 423; Tuck & Ree, 2013, 647–48). This articulation of desire is reflected in my interlocutors’ assertive commitment to a lifeworld that encompasses relational and ecological domains of care, a commitment that contrasts with stereotyped accounts of immiseration, damage, and decline (see Tuck, 2009; Million, 2020; Grande, 2021; Rountree & Smith, 2016; Hobart & Kneese, 2020; Niezen [1998] 2009). The intimate praxis of resurgent care is thus illustrative, in Million's terms, of “resurgence” as “everyday acts of life that communities strive for” (Million, 2020, 394).

## CONCLUSION

The last two decades have witnessed a profusion of scholarship on care within medical anthropology (e.g., Kleinman, 2009, 2019; Biehl, 2013; Garcia, 2010; Han, 2012; Stevenson, 2014; Aulino, 2016; Sadruddin, 2022, 2020). While offering nuanced accounts of the moral, emotional, spiritual, and physical significance of everyday caregiving, this body of scholarship has powerfully illuminated the ways in which practices and relations of care can become intertwined with physical and psychosocial harm, interpersonal and state violence and neglect, and spiritual and material dispossession. The analytic of resurgent care amidst ecosocial injury advances this scholarship by foregrounding the intimate praxis of care as a vital site of agentic aspirations through which my interlocutors strive to “remake a world” (Das et al., 2001; Hobart & Kneese, 2020) in the face of pain and precarity—particularly the effects of Nordic postwar state‐building and assimilation on kin and ecological relations of care in Sápmi. Moreover, my longitudinal ethnographic analysis builds upon and reaffirms critical insights from an interdisciplinary body of scholarship on Indigenous care, resurgence, and kinship (Barker et al., 2020; Grande, 2021; Hobart & Kneese, 2020; Million, 2020; Simpson, 2016). Here, resurgent care represents my interlocutors’ transformative desire to remake, reclaim, and revitalize relational approaches to health, well‐being, and survival. It underscores the radical potentiality of the everyday practices of tending to one another—including more‐than‐human others such as salmon and reindeer.

Recall, in closing, Biret‐Ánne's invocation of “caring for life” (Sá. “*eallima dikšun*”) *qua* caring for people, for language, and for the ecosystem itself—elements to which all are subject, and for whose loss nothing could compensate. As ecological and relational foundations of life become ever more frayed across the globe by crises climatic and political, her account raises a provocation: How might anthropological theorization and praxis expand to illuminate and support the intimate, everyday, transformative care Biret‐Ánne's narrative exemplifies? How might it tend to the life that pulsates through the “main artery” (Sá. “*váldorávdnji*”) of ecologically and intergenerationally embedded relations of care?

## ENDNOTES

1 Állan is a pseudonym. My ethnographic interlocutors have the choice of using pseudonyms or appearing with their real names in my writing, and the opportunity to determine the level of detail in which I may write about what they have shared. (For a more detailed account of my methodological choices and ethnographic process, see Herranen‐Tabibi, 2022a, 7–36; Herranen‐Tabibi, 2022b, 115–19.)

2 Biret‐Ánne is a pseudonym.

3 During my 2 years of continuous fieldwork (2016–2018), altogether 60 interlocutors participated directly in my research (31 female, 29 male; ranging in age from 21 to 80; with 40 residing in Sápmi in Finland, and 20 residing in Sápmi in Norway).

4 See Million (2020) for further analysis of the necessity of holistic approaches to health and well‐being in relation to Indigenous peoples.

5 On caring for memories, see Kleinman (2019).

6 The word “artery” is here used in the sense of a river system's major course or route.
